# Appropriateness of exercise therapy delivery in chronic low back pain management: cross-sectional online survey of physiotherapy practice in Germany

**DOI:** 10.1186/s12891-024-07505-y

**Published:** 2024-05-29

**Authors:** Lukas Kühn, Diane Rosen, Nils Lennart Reiter, Robert Prill, Kyung-Eun (Anna) Choi

**Affiliations:** 1 Center for Health Services Research, Brandenburg Medical School, Seebad 82/83, Rüdersdorf bei Berlin, 15562 Neuruppin, Germany; 2 Faculty of Health Sciences Brandenburg, Brandenburg Medical School, Fehrbelliner Straße 38, 16816 Neuruppin, Germany; 3 Alice Salomon University of Applied Sciences, Alice-Salomon Platz 5, 12627 Berlin, Germany; 4 PhysioBib GbR, Johanniterstraße 26, 10961 Berlin, Germany; 5 Center of Orthopaedics and Traumatology, University Hospital Brandenburg/Havel, Brandenburg Medical School Theodor Fontane, Hochstraße 29, 14770 Brandenburg an der Havel, Neuruppin, Germany; 6 Health Services Research, Medical Imaging and Artificial Intelligence, Faculty of Medicine/Dentistry, Danube Private University, Steiner Landstraße 124, Krems-Stein, 3500 Austria

**Keywords:** Musculoskeletal pain, Low-value care, High-value care, Clinical behavior, Quality of care, Physical therapy, Non-invasive therapy, Movement therapy

## Abstract

**Background:**

In Germany, exercise therapy represents the most commonly prescribed physiotherapy service for non-specific, chronic low back pain (NSCLBP). So far, little is known about current practice patterns of German physiotherapists in delivering this intervention. Thus, the aim of this study was to investigate the appropriateness of exercise therapy delivered to NSCLBP patients in German physiotherapy care and to identify practitioner-related drivers of appropriate exercise delivery.

**Methods:**

We used a vignette-based, exploratory, cross-sectional, online-survey study design (76-items; data collection between May and July 2023). Eligible participants were required to hold a professional degree in physiotherapy and were required to be practicing in Germany. Access links to anonymous online surveys were spread via established German physiotherapy networks, educational platforms, social media, e-mail lists, and snowball sampling. Appropriateness of exercise therapy was calculated by an equally weighted total score (400 points) including scales on shared-decision-making, exercise dose selection, pain knowledge and self-management promotion. “Appropriate exercise delivery” was determined by a relative total score achievement of > 80%. “Partly appropriate exercise delivery” was determined by a relative total score achievement of 50–79%, and “inappropriate exercise delivery” by a score achievement of < 50%. Practitioner-related drivers of exercise appropriateness were calculated by bivariate and multiple linear regression analyses.

**Results:**

11.9% (*N* = 35) of 298 physiotherapists’ exercise delivery was considered “appropriate”, 83.3% (*N* = 245) was “partly appropriate”, and 4.8% (*N* = 14) was “inappropriate”. In the final multiple regression model, most robust parameters positively influencing appropriate delivery of exercise therapy were increased scientific literacy (B = 10.540; 95% CI [0.837; 20.243]), increased average clinical assessment time (B = 0.461; 95% CI [0.134; 0.789]), increased self-perceived treatment competence (B = 7.180; 95% CI [3.058; 11.302], and short work experience (B = − 0.520; 95% CI [-0.959; − 0.081]).

**Conclusion:**

Appropriate exercise delivery in NSCLBP management was achieved by only 11.9% of respondents. However, the vast majority of 95.2% of respondents was classified to deliver exercise therapy partly appropriate. Long work experience seemed to negatively affect appropriate exercise delivery. Positive influences were attributed to scientific literacy, the average clinical assessment time per patient as well as the perceived treatment competence in NSCLBP management.

**Registration:**

Open science framework: 10.17605/OSF.IO/S76MF.

**Supplementary Information:**

The online version contains supplementary material available at 10.1186/s12891-024-07505-y.

## Introduction

### Background

Exercise therapy is recognized as an evidence-based treatment approach in non-specific, chronic low back pain (NSCLBP) management [1]. Compared to no treatment, usual care, or placebo it is associated with meaningful improvements in pain outcomes (MD -15.2, 95% CI -18.3 to -12.2) and functional limitations outcomes (MD -6.8, 95% CI -8.3 to -5.3) [2]. In German physiotherapy (PT) care, exercise therapy represents the most commonly prescribed and delivered service (51.0%) and is responsible for 41.0% of the total turnover of physiotherapeutic services [3]. To this date, it however remains unclear, how exercise therapy is delivered to its individual consumers and whether the delivery is aligned to best available evidence recommendations.

Due to an inconsistency in study designs and insufficient regime descriptions, recommendations on the type (i.e., aquatic exercises, stretching, back schools, McKenzie exercise approach, yoga, or tai-chi) and how to deliver exercise regimes (i.e., individually designed programs, supervised home exercise, or group exercise) remain a point of critical discussion and vary across clinical practice guideline recommendations [1]. However, a lately published comprehensive review reflecting on recent systematic reviews, meta-analyses, guideline recommendations and high-quality randomized controlled trials gives guidance on how to deliver exercise therapy appropriately [4]. According to Cashin and colleagues [4], domains associated with appropriate exercise delivery comprise “clinical assessment”, “exercise type”, “exercise dose”, and “exercise promotion”. Key aspects of an evidence-informed clinical assessment include a comprehensive red flag screening for sufficient exclusion of serious spinal pathologies [5]. Additionally, a detailed history, pain and physical assessment is recommended to assist the clinical practitioner in the development of an exercise regime [4]. This addresses the biopsychosocial patient profile, individual preferences, exercise experiences, and contextual factors [4] and is negotiated within a process of shared-decision making [6, 7]. With regards to exercise type selection, a range of exercise regimes have shown to be effective, including yoga [8], motor control exercise [9, 10], Pilates [9, 11], Tai Chi [12], graded activity [13], aerobic and resistance exercise [9, 14].

Considering the choice of appropriate exercise dosage, evidence from a meta-analysis suggests exercise interventions of 8 to 12 weeks duration with 20 h or more of total exercise engagement [15]. Moreover, the majority of available evidence is based on studies applying an average duration of 30 to 45 min for a single exercise session which is executed at least 2 times per week [14, 16], using a low to moderate intensity [17]. For all regimes, a gradual increase of loads and volumes incorporating concepts of graded exposure or progressive overload may promise added value to achieve patient-relevant therapy goals [4]. To extrapolate and sustain exercise-associated effects, exercise promotion interventions have shown to be beneficial: Aspects of enhancement incorporate exercise supervision containing regular real-time instruction, encouragement, reassurance and adequate progression of exercise dosage [18]. Effective pain education should reassure, foster self-management, support positive coping strategies and eliminate fear and uncertainty about exercise and pain exacerbation [19, 20]. The facilitation of patient-led goal setting practices is associated with improved exercise adherence, self-efficacy and motivation [20, 21].

In Germany, studies investigating appropriate exercise delivery are not available [22]. However, Bahns et al. [23] identified that resistance exercise is the most commonly applied regime across German physiotherapists in NSCLBP management. According to the latest report (2021/2022) of a German statutory health insurance company, it is estimated that each insured patient who claimed PT care, received an average of 19.5 services per year [3]. In order to understand exercise delivery for chronic LBP patients in German PT comprehensively, the following objective was stated:

### Objective

The primary aim of this online survey was to investigate behavior patterns reflecting on the appropriateness of exercise therapy delivered to NSCLBP patients by German physiotherapists. Additionally, practitioner-related drivers affecting the appropriateness of care were aimed to be identified. At the time of study preparation, the authors hypothesized that exercise therapy for NSCLBP patients in German PT care was mainly delivered inappropriately. Thus, this study was led by the following research questions:

1. Are behavior patterns of German physiotherapists indicating appropriate exercise delivery to NSCLBP patients?
2. What are practitioner-related drivers affecting appropriate exercise therapy to NSCLBP patients?

### Methods

This online-survey study was conducted by an interdisciplinary working group with proven expertise in PT, health services research and psychology. Ethical consultancy was ensured by the ethics committee of the Brandenburg Medical School. For this study, a waiver for ethical approval was claimed as data selection was executed anonymously (E-01-20221124). However, data management was guided by the highest data protection standards as data was stored on encoded server files of the Brandenburg Medical School with exclusive access by the research team. To guarantee high-quality reporting standards, the reporting of this study was led by the CHERRIES checklist for online surveys [24] which is provided in Appendix I. A priori study registration was conducted on Open Science Framework [25].

### Setting

In Germany, PT service provision is established in secondary care. In that respect, physicians incorporate a gatekeeper role by holding the authority to prescribe the type and volume (number of treatment sessions and its weekly frequency) of PT services [26]. Moreover, German occupational legislation prohibits physiotherapists to perform medical diagnostics, to provide invasive therapy techniques, or to use manipulative manual therapy approaches [26]. The types of prescribed PT services are to be understood as interventional prescription groups. Most commonly prescribed intervention groups are represented by functional exercise therapy, manual therapy, massage therapy, neurological therapy, or device-based medical resistance training [26]. Within intervention groups, physiotherapists act independently in their individual therapy design [26]. Lately, an introduction of blank prescriptions allowing therapists to personally decide on the type and volume of provided PT services is under current negotiation by German policy makers [27]. The German PT education system is mostly non-academic and takes place in vocational schools. In 2015, estimates indicated that 2.3% of German physiotherapists held an academic degree [28].

### Study design

This cross-sectional, observational study was designed as a nationwide, open web survey. Data were collected between May and July 2023. Prior to the survey start, study participants received written information about survey aims, approximated duration and data protection actions. Subsequently, study participants gave informed consent for data analysis and publication of disclosures made before beginning the survey. During the full process, participation was held anonymously as an implementation of cookies, or an IP-address check was disclaimed making duplicate checks infeasible. Study participation was voluntary and has not been compensated.

### Data collection

Participants were eligible if they were clinically working as a physiotherapist in Germany and at least 18 years of age. A convenience sampling strategy was applied to recruit eligible participants. Data were collected via established PT networks (www.physio.de; www.physiobib.de) and social media (www.facebook.de). On social media, advertisements were actively placed and based on a tailored filter system targeting platform users with interests in PT. Within PT networks, advertisements were posted on online forums and Instagram channels of targeted networks. In addition, a professional association’s practice locator tool (www.physio-deutschland.de) was used to manually create an email list to directly contact physical therapy practices. Participants were additionally invited to distribute the survey among their peer groups. As this study was explorative, we dispensed on calculating a sample size.

The questionnaire was developed and distributed in German language by using the online application software Limesurvey (Hamburg, Germany). The average editing time for questionnaire completion was estimated to take 15 min. In case of incomplete questionnaires, participants were reminded to complete missing questions before questionnaire submission. To avoid bias within the editing process, answered questions were not able to be altered at a later point in time. Due to a sequential series of survey items, randomization of items was not feasible. The questionnaire was presented via eight screens. The number of items presented per screen ranged from one to 18.

### Questionnaire development and pre-testing

The questionnaire was specifically designed for this study. Thematically, it based on a narrative literature review on delivery formats of exercise therapy for patients suffering NSCLBP conditions [4]. The final questionnaire version was structured in four thematic domains consisting of (a) type of exercise selection (including shared decision making); (b) exercise dose selection (including frequency, intensity, volume, scope); (c) exercise promotion (including knowledge about pain mechanisms, and self-management promotion), and (d) participant characteristics (including demographic characteristics, work-related characteristics and 16 items of the German Version of the Tampa Scale of Kinesiophobia for Physiotherapists (TSK-PT) [29]). Although the domain of “clinical assessment” has also been described to influence appropriate exercise delivery [4], we relinquished to include this domain in the questionnaire as investigations referring to clinical assessment behaviors in NSCLBP patients have previously been conducted in Germany [23]. Face validity was tested by piloting the first version of the questionnaire via face-to-face consultations among five clinically working physiotherapists and five researchers. Participants of the pilot-testing phase were asked to highlight complications with theoretical considerations, understandings, semantics, or layout conditions. Written feedback was provided by using a pre-developed feedback sheet.

### Vignette

To investigate domains (a), (b) and (c), applied questionnaire items referred to a validated case vignette [30] describing an NSCLBP patient. By conducting a feedback and consensus process (LK, AC; DR; NR; RP), the validated case vignette was further adapted and tailored to specific legislative PT care conditions in Germany. The applied vignette consisted of two consecutive parts: Part A referred to domain (a) type of exercise selection. Part B was developed to tailor the case scenario to one specific exercise regime. In that respect, we decided to focus on a resistance exercise regime as this represents the most commonly applied regime among German physiotherapists [23]. Part B of the vignette was followed by questionnaire items referring to domains (b) exercise dose, and (c) exercise promotion. The final vignette is described in Table 1.

**Table 1:** Description of the case vignette

**Part A**
Lisa, 35, is referred to physical therapy by her primary care physician after suffering from severe low back pain for 16–18 weeks.
In the past few years, she has not had the energy to be physically active. She has been on sick leave from her job as a healthcare assistant since the episode started.
This is the third and worst episode of low back pain she has experienced. In the two previous episodes, the pain has resolved spontaneously. The pain is currently reduced to approximately 50% of its worst intensity during this episode. The pain does not disturb her sleep. She is currently taking paracetamol.
She is very concerned about the intensity of the pain and she is nervous that her back problems will not resolve this time. Lisa feels she still needs to rest her back once in a while. She is afraid of exacerbating the pain again, in case she has to lift something from an awkward position
Diagnostic report of Lisa’s primary care physician: The neurological examination is normal. The MRI scan shows age relate degenerative changes of the lumbar spine. Serious spinal pathologies were ruled out. Diagnosis: “Unspecific, chronic low back pain”.
The primary care physician has written a prescription for exercise therapy. Regarding the type and volume of exercise therapy, the physician expressly seeks your physiotherapeutic expertise.
**Part B**
You agreed with Lisa on an exercise program that focuses resistance training. You also agreed with Lisa on the following treatment goals: Improvement of general physical function and performance. Pain reduction and increased exercise tolerance. Improvement of general psychological well-being.

### Measuring appropriateness of exercise therapy

To measure appropriateness of exercise therapy, validated and self-developed scales were combined. With regards to domain (a) “type of exercise”, a stand-alone categorical, multiple response item (multiple choice format) was used to explore which evidence-based exercise approaches (Pilates, Yoga, Graded Activity, resistance training, Motor Control, Tai Chi, endurance training, other) were regularly used by respondents. This item was descriptive in nature and was not integrated in the final score reflecting on the appropriateness of exercise therapy. Moreover, the validated German version of the 9-item shared-decision-making-questionnaire for physicians (SDM-Q-Doc) was applied to explore the selection process of appropriate exercise interventions for the individual patient [31]. Survey items reflecting on domain (a) “type of exercise” appeared in the questionnaire after part A of the case vignette was introduced.

In the next step, part B of the case vignette was introduced. Considering domain (b) “dose”, four categorical, single response items were developed to explore survey respondents’ delivery of resistance exercise (Part B of case vignette) under circumstances of the written case scenario. Applied items reflected on the frequency (recommended number of training sessions per week), intensity (BORG Scale ranging from 6 to 20), volume (minutes per therapy session), and scope (time period of therapy per weeks) of applied exercise regimes. For each item, one score-point was assigned if the response was in line with therapy recommendations [4]. Response options were provided in a multiple choice format. At maximum, a total score of four points could be achieved.

Domain (c) “exercise promotion” included the sub-domains “knowledge about pain mechanisms” and “self-management promotion”. Knowledge about pain mechanisms was explored using the validated 12-item German version of the neurophysiology of pain questionnaire (NPQ-D) [32]. The promotion of self-management capabilities was measured by a self-developed, non-validated 11-item scale (SMP-S) as we did not consider available self-management promotion scales being suitable to this specific study. This 11-item score based on a five-point-likert scale ranging from 0 “never” to 4 “always” resulting in a maximum number of 44 points to be achieved. Self-developed survey items and its point-based score systems are illustrated in Appendix II. Survey items of applied validated scales and their methods of analysis are described elsewhere [29, 31, 32]. For each of the four highlighted scales, an equally weighted score was calculated by a linear transformation of scales from 0 to 100. In this respect, transformed scores were combined and aggregated, resulting in a possible maximum score of 400 points per survey respondent.

### Data analysis

Collected Limesurvey data were exported and analysed via IBM SPSS Statistics Software Version 23 (Armonk, New York, United States). As available imputation methods underlie serious concerns of representativeness, cases with missing values were excluded from analyses. To analyse the appropriateness of exercise therapy, descriptive statistics were used by reporting distributions of weighted sub-scores and the aggregated, weighted total score. For self-developed scores (dose and SMP-S) frequencies of responses of single-score items were additionally reported. Delivery of exercise therapy was considered appropriate by calculating a set threshold of 80% of total score achievement. Delivery of exercise therapy was considered partly appropriate by a range of 50–79% threshold achievements. Inappropriate exercise delivery was determined by a relative score achievement of < 50%. This range of thresholds has been selected as it has been the point of reference in previous studies investigating guideline adherence in acute or chronic low back pain (LBP) management [23, 33, 34]. Associations between the appropriateness of exercise therapy and provider characteristics were calculated by exploratory, stepwise, univariate linear regression models informing a final multiple linear regression model. B-coefficients, standard errors, Beta, 95% CI, and p-values were reported for the final regression table. The level of statistical significance was set at *p* ≤ .05. Provider characteristics included a pre-defined set of sociodemographic and work-related characteristics. Prior to regression modelling, independent discrete variables were tested for multicollinearity using the Spearman correlation coefficient *r*. Normal distribution of residuals was tested by the Shapiro Wilk test. Homoscedasticity was checked graphically by creating a scatterplot and q-q-plot of residuals.

## Results

### Sociodemographic and professional characteristics

In total, we received 509 responses of which 298 participants (58.5%) completed the survey. Of the 298 participants, 195 (65.7%) were female. The mean age of included participants was 45.2 years (SD = 13.6 years) and participants reported an average work experience of 21.2 years (SD = 13.4). 277 (93.3%) of all respondents worked in outpatient PT practice settings and 177 (59.6%) reported to be self-employed. 259 (87.5%) participants had a residency in West Germany compared to 37 (12.5%) participants residing in East Germany (including Berlin). 58 participants (19.5%) had undergone an academic physiotherapeutic education program. The most frequently completed professional training courses represented manual therapy (*N* = 199 (66.8%)) and device-based medical resistance training (*N* = 157 (52.7%)). 150 (51.4%) respondents perceived their personal treatment competences in NSCLBP management to be above average. The average number of NSCLBP patients personally treated per week was 11.2 (SD = 16.7). A comprehensive display of sociodemographic and work-related characteristics of survey participants is provided in Table 2.

**Table 2:** Sociodemographic and work-related characteristics of participants (*N* = 298)

Characteristics	Values
Age (years)	45.2 (13.6)
**Gender**	
*Female*	195 (65.7)
*Male*	101 (34.0)
*Diverse*	1 (0.3)
**Work setting**	
*Outpatient practice*	277 (93.3)
*Hospital*	4 (1.3)
*Rehabilitation clinic*	3 (1.0)
*Other*	13 (4.4)
**Employment**	
*Self-employed*	177 (59.6)
*Employed*	105 (35.4)
*Freelance*	15 (5.1)
Colleagues (number)	9.5 (40.2)
*Working time (hours per week)*	32.8 (12.6)
*Work experience (years)*	21.2 (13.4)
**Regular exchange with other professions** ^**a**^	
*Medicine*	150 (50.3)
*Psychology*	31 (10.4)
*Nursing*	57 (19.1)
*Occupational therapy*	51 (17.1)
*Other*	72 (24.2)
**Highest professional degree**	
*Diploma (vocational school)*	239 (80.5)
*Bachelor (university)*	44 (14.8)
*Master (university)*	12 (4.0)
*Doctorate*	2 (0.7)
**Performed training courses** ^**a**^	
*Manual therapy*	199 (66.8)
*Orthopaedic manual therapy (OMT)*	30 (10.1)
*Osteopathy*	47 (15.8)
*Pain management therapy*	34 (11.4)
*Naturopath*	85 (28.5)
*Device-based medical resistance training*	157 (52.7)
*Other*	215 (72.1)
**Information sources** ^**a**^	
*Scientific journals*	208 (69.8)
*Clinical practice guidelines*	138 (46.3)
*Information sources from professional associations*	122 (40.9)
*Collegial exchange of experiences*	201 (67.4)
*Professional training courses*	237 (79.5)
*Other*	92 (30.9)
**Membership in professional association**	
*Yes*	216 (72.7)
*No*	81 (27.3)
NSCLBP patients treated at facility (number per week)	34.8 (36.9)
Estimated average clinical assessment time (minutes)	21.5 (13.7)
Kinesiophobia (16 items of TSK-PT-G)	33.21 (9.20)
Rating of personal NSCLBP treatment competence	
*Above average*	150 (51.4)
*Neutral*	92 (31.5)
*Below Average*	50 (17.1)

*Legend* Categorical variables are expressed as number (%); Continuous variables are expressed as Mean (SD); ^a^multiple response option; TSK-PT-G German version of the Tampa Scale for Kinesiophobia for Physiotherapists (16 items used)

### Appropriateness of exercise therapy

With regards to domain (a) “type of exercise”, 143 participants (48.0%) reported to routinely use resistance training. This was followed by Yoga (*N* = 133 (44.6%)), endurance training (*N* = 119 (39.9%)), and Graded Activity (*N* = 117 (39.3%)). The least frequently used exercise regimes were Tai Chi (*N* = 44 (14.8%)), Motor Control training (*N* = 71 (23.8%)), and Pilates (*N* = 109 (36.6%)). Additionally, 129 participants (43.3%) stated to use other than listed exercise regimes. Considering a shared-decision-making approach in the selection of appropriate exercise regimes for NSCLBP patients, the mean score of the weighted SDM-Q-Doc scale was at Mean = 79.28 (SD = 14.53).

**Fig. 1:**
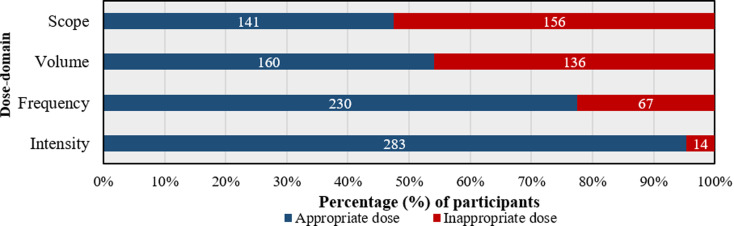
Appropriateness of applied exercise dose in reference to the case vignette. *Legend* Scope: *Over what period of time should the exercise therapy be practiced at minimum?* (appropriate scope: 12 weeks); Volume: *How many minutes should one training session consist of at minimum?* (appropriate volume: 30 to 45 min); Frequency: *How many times per week should Lisa perform resistance training at minimum?* (appropriate frequency: 2 to 3 times per week); Intensity: *What should be the training intensity as perceived by Lisa?* (appropriate intensity: Borg scale 11 to 16)

Considering domain (b) “exercise dose” for resistance training (referring to Part B of the written case vignette), 230 respondents (77.4%) selected an appropriate frequency of two to three therapy sessions per week. 283 respondents (95.3%) selected an appropriate training intensity which includes a patient-led perceived perception of exertion scale values ranging from 11 to 16. 160 respondents (54.1%) selected an appropriate volume of 30 to 45 min for each training session and 141 respondents (47.5%) selected an appropriate minimal scope of exercise therapy of twelve weeks. Taking the weighted score on the appropriateness of exercise dose into account, 69 participants (23.2%) reached the maximum point-score of 100. Descriptive statistics on survey items reflecting on sub-domains of exercise dose are provided in Fig. 1.

**Fig. 2:**
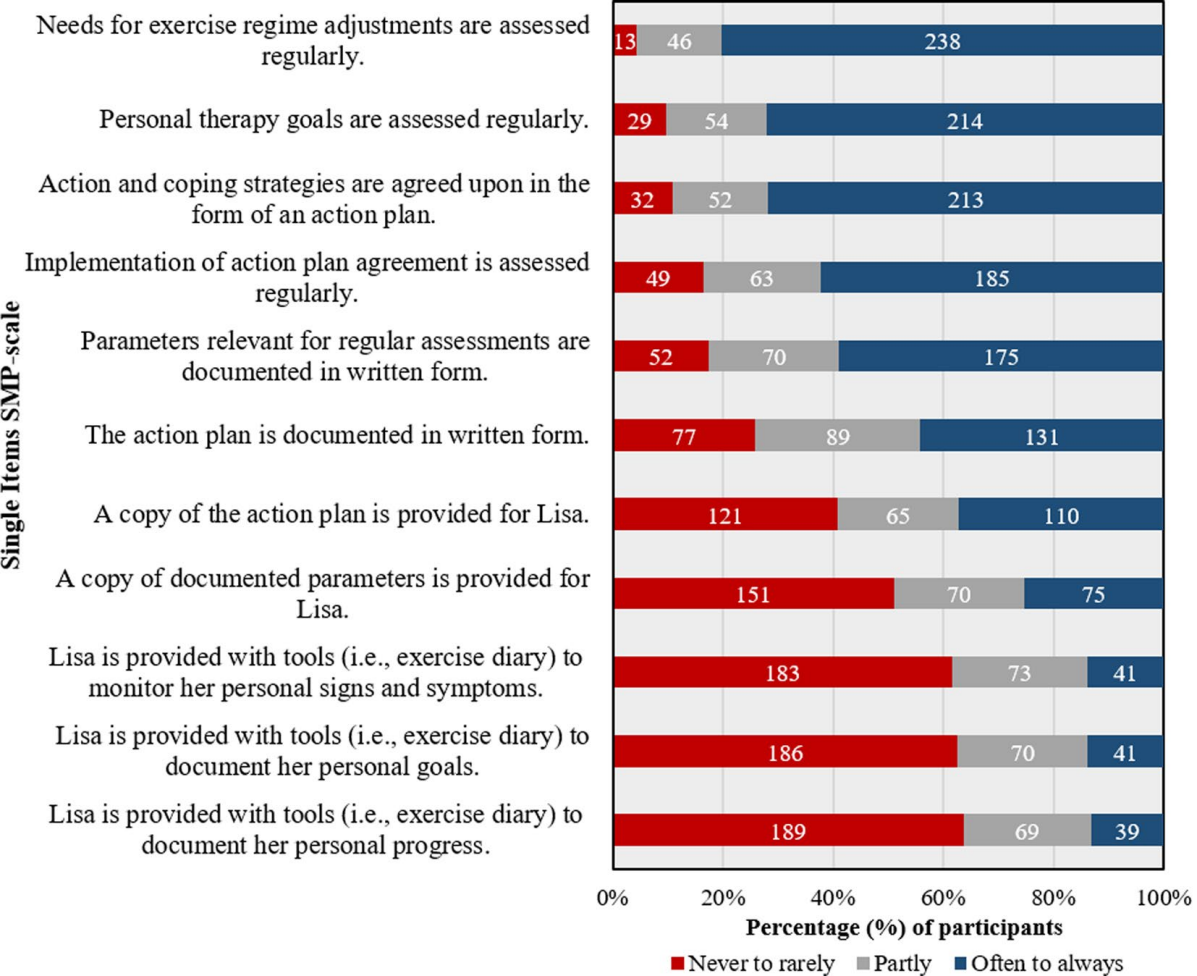
Illustration of single item-responses in descending order of self-developed self-management-promotion-scale (SMP) (*N* = 298)

In domain (c) “exercise promotion”, the mean score of the weighted NPQ-D was at Mean = 68.60 (SD = 17.93). For the SMP-S, the mean number of achieved points was at Mean = 53.26 (SD = 18.86). A detailed illustration of items referring to the SMP-S is provided in Fig. 2. The combination of individual scales into a total score revealed a range of total points achieved of Minimum = 149.09 points to Maximum = 375.00 points (Median = 269.95 points). Total score calculation could be performed for *N* = 294 participants. In that respect, 11.9% (*N* = 35) of respondents delivered exercise therapy appropriately. 83.3% (*N* = 245) delivered exercise therapy partially appropriate, and 4.8% (*N* = 14) of respondents delivered exercise therapy inappropriately.

### Drivers of appropriate exercise delivery

For the final multiple, linear regression model, significant Spearman’s *r* correlations of independent, discrete variables ranged from *r* = .115 to *r* = .189. The Shapiro Wilk test of standardized residuals demonstrated a level of significance of *p* = .407. To further demonstrate normal distribution and homoscedasticity of residuals, a histogram; q-q-plot and scatterplot of standardized residuals is provided in Appendix III. For the final model, significant independent variables on the total score of exercise appropriateness were “work experience” (B = − 0.520; 95% CI [-0.959; − 0.081]; *p* = .020), “scientific journals regularly used as information sources” (B = 10.540; 95% CI [0.837; 20.243]; *p* = .033), “membership in a professional association” (B = 13.933; 95% CI [0.533; 27.433]; *p* = .042), “average clinical assessment time” (B 0 0.461; 95% CI [0.134; 0.789]; *p* = .006), and “perceived personal treatment competence” (B = 7.180; 95% CI [3.058; 11.302]; *p* = .001). Table 3 illustrates the final multiple, linear regression model which was informed by significant bivariate linear regression models.

**Table 3:** Associations between participant characteristics and appropriate exercise delivery

	B	Std. error	Beta	95% CI of B	*p*-value
**Bivariate linear regression models**					
Work experience (years)	− 0.562	0.174	− 0.186	[-0.905; − 0.219]	0.001
**Employment**					
*Employed*	Reference	Reference	Reference	Reference	Reference
*Self-employed*	-11.852	4.915	− 0.140	[-21.525; -2.179]	0.017
**Professional degree**					
*Non-academic*	Reference	Reference	Reference	Reference	Reference
*academic*	23.132	5.808	0.227	[11.701; 34.563]	0.000
**Scientific journals are regular sources of information**					
*No*	Reference	Reference	Reference	Reference	Reference
*Yes*	18.014	5.057	0.204	[8.062; 27.967]	0.000
**Member in professional association**					
*No*	Reference	Reference	Reference	Reference	Reference
*Yes*	-2.270	5.331	− 0.025	[-12.762; 8.222]	0.671
Weekly treated number of NSCLBP patients at facility	0.177	0.065	0.162	[0.050; 0.304]	0.006
Average clinical assessment time (minutes)	0.576	0.170	0.194	[0.241; 0.911]	0.001
Self-perceived treatment competence (7-point-likert-scale)	8.649	2.067	0.239	[4.581; 12.718]	0.000
Kinesiophobia (TSK-PT-G)	− 0.933	0.253	− 0.213	[-1.431; − 0.433]	0.000
**Multiple linear regression model**					
Work experience (years)	− 0.520	0.223	− 0.173	[-0.959; − 0.081]	0.020
**Employment**					
*Employed*	Reference	Reference	Reference	Reference	Reference
*Self-Employed*	-8.987	6.912	− 0.106	[-22.597; 4.622]	0.195
**Professional degree**					
*Non-academic*	Reference	Reference	Reference	Reference	Reference
*Academic*	9.226	6.120	0.091	[-2.824; 21.277]	0.133
**Scientific journals are regular sources of information**					
*No*	Reference	Reference	Reference	Reference	Reference
*Yes*	10.540	4.928	0.119	[0.837; 20.243]	0.033
**Member in professional association**					
*No*	Reference	Reference	Reference	Reference	Reference
*Yes*	13.933	6.836	0.154	[0.533; 27.433]	0.042
Weekly treated number of NSCLBP patients at facility	0.110	0.061	0.101	[-0.010; 0.231]	0.073
Average clinical assessment time (minutes)	0.461	0.166	0.155	[0.134; 0.789]	0.006
Self-perceived treatment competence (7-point-likert-scale)	7.180	2.094	0.201	[3.058; 11.302]	0.001
Kinesiophobia (TSK-PT-G)	− 0.470	0.257	− 0.106	[0.134; 0.789]	0.069
**Constant**	236.416	14.258	N/A	[208.344; 264.488]	0.000

*Legend* Dependent variable: Total score on exercise appropriateness; Multiple linear regression model: R square = 0.204; Adjusted R square = 0.177.

## Discussion

To the best of our knowledge, this is the first study conducted on German physiotherapists exploring delivery patterns of exercise therapy in NSCLBP conditions. Considering shared-decision-making endeavors in the selection process of individual exercise regimes, appropriate dose selection, as well as exercise promotion endeavors including knowledge about pain and self-management promotion, 11.7% of the survey sample reached the 80% threshold indicating appropriate exercise delivery. Long work experience seemed to negatively affect appropriate exercise delivery. Positive influences were attributed to scientific literacy, a membership in a professional association, the average clinical assessment time per patient as well as the perceived treatment competence in NSCLBP management.

Our results are not in line with comparable findings. In a previously mentioned study of Bahns and colleagues [23], guideline adherence to LBP treatment of German physiotherapists was also measured by an online survey and determined by an 80% threshold of adherence. The authors identified that 72% of respondents adhered to LBP treatment recommendations of the German National Guideline on LBP (NVL) [35]. This is opposed to 11.7% of this study sample considered to deliver exercise therapy appropriately. One reason of this difference in observation may be explained by the fact that the NVL rather provides recommendation on treatment options than on specific delivery formats for each of them [35]. However, in line with the findings of Bahns and colleagues [23], resistance training represented the preferred exercise regime of surveyed respondents.

In another vignette-based survey study of current PT practice patterns in LBP management, the authors identified that Canadian physiotherapists preferred to treat LBP patients two to three times per week, for 30 to 60 min per session, over a time period of one to three months [36]. These findings of Orozco and colleagues [36] are consistent with answers to the dose selection process of our study. In comparison, a survey study conducted in New Zealand revealed that physiotherapists regularly provided between six to ten treatment sessions for NSCLBP patients and highlighted that this scope of therapy is not sufficient to support patients in self-managing their chronic conditions [37].

Considering the shared-decision-making process as a prerequisite to successfully select appropriate exercise regimes for NSCLBP patients, respondents of this study received rather high score values on the SDM-Q-Doc. In a systematic review on the use of the SDM-Q-Doc and the 9-item shared-decision-making questionnaire for patients (SDM-Q-9), a mean score between 42 and 75 points was reported. This is lower than the observed mean score of this study [38]. Possibly, the relevance of the concept of shared-decision-making has evolved since 2017 and explains relatively high score results. Regarding the mean score of the original 12-point-scale of the NPQ-D, respondents of this survey reached comparable scores-values (Mean = 8.23 ± 2.15) to respondents of the German validation article of the NPQ-D (Mean = 9.34 ± 1.88) [32].

In a study on the use and acceptance of LBP guidelines among physiotherapists in New Zealand, higher LBP caseloads and higher professional degrees were positively associated with the perception of guidelines being helpful in clinical decision-making processes [39]. These findings are in line with our results in which a higher professional degree and a higher LBP caseload on facility-level showed positive, univariate associations with increased total score values on exercise appropriateness. However, these associations faded in the multiple regression model of our study as well as in the model of Hendrick and colleagues [39].

In our model, strongest predictor variables for appropriate exercise therapy were work experience, scientific literacy, average clinical assessment time and self-perceived treatment competence. With regards to work experience, a review on physicians’ guideline adherence in cardiology supports our finding that older age and increased work experience is associated with lower levels of adherence [40]. However, a survey study on Nigerian physiotherapists’ guideline adherence in LBP management did not identify an association of age and guideline adherence in any direction [33].

In our analysis, higher numbers of respondents’ average clinical assessment time were positively associated with increased total score values on appropriate exercise delivery. This finding is supported by an experimental study conducted among primary care physicians which revealed that under time pressure, guideline adherence and especially relevant aspects of history taking and advice giving were compromised [41]. These findings are also supported by qualitative investigations on the appropriateness on diagnostic imaging in LBP conditions in which general practitioners and radiologists reported time restrictions to represent major barriers in guideline concordant imaging procedures [42].

Opposed to our finding that the self-perceived treatment competence indicates actual knowledge and skills in exercise delivery for NSCLBP management, Griffin and colleagues [43] did not identify this mechanism in surveying Irish physiotherapists to rate their competence in nutrition care. Possibly, this divergence might be explained by the fact that nutrition competencies do not represent a standard educational building block of curricula in PT education.

## Limitations

There are limitations to state. This study was cross-sectional in its design and does not allow any conclusions on cause and effect relationships. Moreover, common recruitment strategies (i.e., social media, e-mail lists) of online-surveys as conducted in this study, underlie concerns of representativeness [44]. We cannot fully comprehend who came into contact with this survey, and we assume that respondents with an above-average interest into the topic are dominant in the sample. We are therefore cautious to project our results to the general population of German physiotherapists. Specific to this sample, we noticed an overrepresentation of respondents with university degrees, or with long professional work experiences, or with self-employed employment status. On the other hand, we observed an underrepresentation of respondents with residency in East Germany or of respondents working in inpatient care settings.

To explore and investigate clinical behavior, we used a vignette-based survey study design. Conducted inappropriately, this approach is criticized to not sufficiently represent real-world phenomena which can raise concerns of internal and external validity [45]. To counter this risk, we relied on a vignette of an unspecific, NSCLBP case that has already been tested [30]. As we tailored this case scenario to legislative conditions of PT care in Germany, we additionally tested face validity of the final vignette among a group of researchers and practitioners. We thus assume a high reliability of the used case scenario to appropriately represent a patient scenario of unspecific, NSCLBP even though we did not follow a rigorous cultural adaptation process as described by Beaton and colleagues [46].

A clear limitation to state is the applied self-developed scale reflecting on self-management promotion (SMP-S). However, its development followed a literature informed process in which seven quality indicators of the Evidence Summary (JBI-ES-1295-3) on self-management in chronic diseases (Joanna Briggs Institute, Adelaide, Australia) were integrated [47]. We therefore assume that the SMP-S reliably reflects relevant aspects of self-management promotion in chronic disease management.

For aims of this study, thresholds distinguishing between appropriate (80% total score achievement), partly appropriate (50–79% total score achievement) and inappropriate (< 50% total score achievement) exercise delivery were determined. By doing so, we relied on previously conducted studies which followed comparable aims and conditions [23, 33, 34]. However, if this discrimination of PT service delivery actually reflects the best classification of appropriate versus inappropriate service delivery remains to be answered.

As previously mentioned, performing an appropriate diagnostic process represents an elementary prerequisite prior to planning and delivering exercise therapy in NSCLBP patients [4]. In order to minimize the burden of study participants, we have not been able to include this aspect of PT service delivery into our questionnaire. However, in-depth investigations on diagnostic practice patterns of German physiotherapists in LBP conditions have recently been reported [23].

## Conclusion

Appropriate exercise delivery in NSCLBP management was achieved by 11.9% of respondents. However, the vast majority of 95.2% of respondents was classified to deliver exercise therapy partly appropriate. Most relevant provider-centric drivers of appropriate exercise delivery were work experience, scientific literacy, the average clinical assessment time, and the self-perceived treatment competence of respondents.

### Electronic supplementary material

Below is the link to the electronic supplementary material.

Supplementary Material 1. Appendix I

Supplementary Material 2. Appendix II

Supplementary Material 3. Appendix III

## Data Availability

The datasets used and analyzed during the current study are available from the corresponding author on reasonable request.

## Abbreviations

LBP: non-specific, acute or chronic low back pain
NSCLBP: non-specific, chronic low back pain
PT: physiotherapy

## References

[1] Oliveira CB, Maher CG, Pinto RZ, Traeger AC, Lin C-WC, Chenot J-F (2018). Clinical practice guidelines for the management of non-specific low back pain in primary care: an updated overview. Eur Spine J.

[2] Hayden JA, Ellis J, Ogilvie R, Malmivaara A, van Tulder MW. Exercise therapy for chronic low back pain. Cochrane Database Syst Reviews. 2021(9). 10.1002/14651858.CD009790.pub2 PMC8477273 34580864

[3] Waltersbacher A, WIDO). Heilmittelbericht 2021/2022 Ergotherapie, Sprachtherapie, Physiotherapie, Podologie Berlin: Wissenschaftliches Institut der AOK (; 2022 [cited 2022 31st August]. https://www.wido.de/fileadmin/Dateien/Dokumente/Publikationen_Produkte/Buchreihen/Heilmittelbericht/wido_hei_heilmittelbericht_2021_2022_final.pdf.

[4] Cashin AG, Booth J, McAuley JH, Jones MD, Hübscher M, Traeger AC et al. Making exercise count: considerations for the role of exercise in back pain treatment. Musculoskelet Care. 2021. 10.1002/msc.1597 34676659

[5] Bardin LD, King P, Maher CG (2017). Diagnostic triage for low back pain: a practical approach for primary care. Med J Aust.

[6] Lin I, Wiles L, Waller R, Goucke R, Nagree Y, Gibberd M (2020). What does best practice care for musculoskeletal pain look like? Eleven consistent recommendations from high-quality clinical practice guidelines: systematic review. Br J Sports Med.

[7] Slade SC, Molloy E, Keating JL (2009). People with non-specific chronic low back pain who have participated in exercise programs have preferences about exercise: a qualitative study. Australian J Physiotherapy.

[8] Wieland LS, Skoetz N, Pilkington K, Vempati R, D’Adamo CR, Berman BM. Yoga treatment for chronic non-specific low back pain. Cochrane Database Syst Reviews. 2017(1). 10.1002/14651858.CD010671.pub2 PMC5294833 28076926

[9] Owen PJ, Miller CT, Mundell NL, Verswijveren SJ, Tagliaferri SD, Brisby H (2020). Which specific modes of exercise training are most effective for treating low back pain? Network meta-analysis. Br J Sports Med.

[10] Saragiotto BT, Maher CG, Yamato TP, Costa LO, Costa LCM, Ostelo RW et al. Motor control exercise for chronic non-specific low‐back pain. Cochrane Database Syst Reviews. 2016(1). 10.1002/14651858.CD012004 PMC8761501 26742533

[11] Yamato TP, Maher CG, Saragiotto BT, Hancock MJ, Ostelo RW, Cabral CM et al. Pilates for low back pain. Cochrane Database Syst Reviews. 2015(7). 10.1002/14651858.CD010265.pub2 PMC8078578 26133923

[12] Hall A, Copsey B, Richmond H, Thompson J, Ferreira M, Latimer J (2017). Effectiveness of tai chi for chronic musculoskeletal pain conditions: updated systematic review and meta-analysis. Phys Ther.

[13] Macedo LG, Smeets RJ, Maher CG, Latimer J, McAuley JH (2010). Graded activity and graded exposure for persistent nonspecific low back pain: a systematic review. Phys Ther.

[14] Wewege MA, Booth J, Parmenter BJ (2018). Aerobic vs. resistance exercise for chronic non-specific low back pain: a systematic review and meta-analysis. J Back Musculoskelet Rehabil.

[15] Hayden JA, Van Tulder MW, Malmivaara AV, Koes BW (2005). Meta-analysis: exercise therapy for nonspecific low back pain. Ann Intern Med.

[16] Geneen LJ, Moore RA, Clarke C, Martin D, Colvin LA, Smith BH. Physical activity and exercise for chronic pain in adults: an overview of Cochrane Reviews. Cochrane Database Syst Reviews. 2017(4). 10.1002/14651858.CD011279.pub3 PMC5461882 28436583

[17] Polaski AM, Phelps AL, Kostek MC, Szucs KA, Kolber BJ (2019). Exercise-induced hypoalgesia: a meta-analysis of exercise dosing for the treatment of chronic pain. PLoS ONE.

[18] Slade SC, Patel S, Underwood M, Keating JL (2014). What are patient beliefs and perceptions about exercise for nonspecific chronic low back pain? A systematic review of qualitative studies. Clin J Pain.

[19] Boutevillain L, Dupeyron A, Rouch C, Richard E, Coudeyre E (2017). Facilitators and barriers to physical activity in people with chronic low back pain: a qualitative study. PLoS ONE.

[20] Nicolson PJ, Bennell KL, Dobson FL, Van Ginckel A, Holden MA, Hinman RS (2017). Interventions to increase adherence to therapeutic exercise in older adults with low back pain and/or hip/knee osteoarthritis: a systematic review and meta-analysis. Br J Sports Med.

[21] Gardner T, Refshauge K, McAuley J, Hübscher M, Goodall S, Smith L (2019). Combined education and patient-led goal setting intervention reduced chronic low back pain disability and intensity at 12 months: a randomised controlled trial. Br J Sports Med.

[22] Peschke D (2019). Bedarfsgerechtigkeit in Der Physiotherapeutischen Versorgung in Deutschland–Ein Scoping Review. Zeitschrift für Evidenz Fortbildung Und Qualität Im Gesundheitswesen.

[23] Bahns C, Happe L, Thiel C, Kopkow C (2021). Physical therapy for patients with low back pain in Germany: a survey of current practice. BMC Musculoskelet Disord.

[24] Eysenbach G (2004). Improving the quality of web surveys: the Checklist for reporting results of internet E-Surveys (CHERRIES). J Med Internet Res.

[25] Kühn L. Appropriateness of exercise therapy in chronic low back pain man-agement: Cross-sectional online survey on physiotherapy practice in Germany. 10.17605/OSF.IO/S76MF. 2023. 10.1186/s12891-024-07505-y PMC11137918 38811932

[26] Brechtel T, Thielscher C (2021). Heil- und Hilfsmittel. Handbuch Medizinökonomie | Grundlagen Und System Der Medizinischen Verorgung.

[27] Verordnungen in der vertragsärztlichen Versorgung Berlin. Gemeinsamer Bundesausschuss; [cited 2023 July 21]. https://www.g-ba.de/themen/veranlasste-leistungen/heilmittel/verordnung-heilmittel-vertragsaerzte/.

[28] Der physiotherapeutische (2017). Direktzugang in Deutschland – Internationaler Vergleich ausbildungsinhaltlicher und struktureller Bedingungen. Physioscience.

[29] Le Laekeman M-A, Sitter H, Basler HD (2008). The Pain attitudes and beliefs Scale for physiotherapists: psychometric properties of the German version. Clin Rehabil.

[30] Husted M, Rossen CB, Jensen TS, Mikkelsen LR, Rolving N (2020). Adherence to key domains in low back pain guidelines: a cross-sectional study of Danish physiotherapists. Physiother Res Int.

[31] Scholl I, Kriston L, Dirmaier J, Buchholz A, Härter M (2012). Development and psychometric properties of the Shared decision making questionnaire–physician version (SDM-Q-Doc). Patient Educ Couns.

[32] Richter M, Maurus B, Egan Moog M (2019). Die deutsche Version Des Neurophysiology of Pain Questionnaire. Der Schmerz.

[33] Akindele M, Rabiu M, Useh E (2020). Assessment of the awareness, adherence, and barriers to low back pain clinical practice guidelines by practicing physiotherapists in a low-resourced country. Physiother Res Int.

[34] Spitaels D, Hermens R, Van Assche D, Verschueren S, Luyten F, Vankrunkelsven P (2017). Are physiotherapists adhering to quality indicators for the management of knee osteoarthritis? An observational study. Musculoskelet Sci Pract.

[35] German national care guideline (NVL). unspecific back pain Berlin: Medical Center for Quality in Medicine (ÄZQ); [cited 2022 31st August]. https://www.leitlinien.de/themen/kreuzschmerz.

[36] Orozco T, Feldman DE, Mazer B, Chilingaryan G, Hunt M, Williams-Jones B (2017). Low back Pain: current patterns of Canadian Physiotherapy Service Delivery. Physiother Can.

[37] Liddle SD, David Baxter G, Gracey JH (2009). Physiotherapists’ use of advice and exercise for the management of chronic low back pain: a national survey. Man Therap.

[38] Doherr H, Christalle E, Kriston L, Härter M, Scholl I (2017). Use of the 9-item Shared decision making questionnaire (SDM-Q-9 and SDM-Q-Doc) in intervention studies—A systematic review. PLoS ONE.

[39] Hendrick P, Mani R, Bishop A, Milosavljevic S, Schneiders AG (2013). Therapist knowledge, adherence and use of low back pain guidelines to inform clinical decisions–a national survey of manipulative and sports physiotherapists in New Zealand. Man Therap.

[40] Hoorn CJGM, Crijns HJGM, Dierick-van Daele ATM, Dekker LRC (2019). Review on factors influencing physician Guideline Adherence in Cardiology. Cardiol Rev.

[41] Evangelia T, Efharis P, Nick S, Anthony M, Alexios B (2013). The influence of time pressure on adherence to guidelines in primary care: an experimental study. BMJ Open.

[42] Gransjøen AM, Wiig S, Lysdahl KB, Hofmann BM (2018). Barriers and facilitators for guideline adherence in diagnostic imaging: an explorative study of GPs’ and radiologists’ perspectives. BMC Health Serv Res.

[43] Griffin A, Conway H, Chawke J, Keane M, Douglas P, Kelly D. An exploration of self-perceived competence in providing nutrition care among physiotherapists in Ireland: a cross-sectional study. Physiother Theory Pract. 2023:1–10. 10.1080/09593985.2023.2243624 37540212

[44] Andrade C (2020). The limitations of online surveys. Indian J Psychol Med.

[45] Evans SC, Roberts MC, Keeley JW, Blossom JB, Amaro CM, Garcia AM (2015). Vignette methodologies for studying clinicians’ decision-making: Validity, utility, and application in ICD-11 field studies. Int J Clin Health Psychol.

[46] Beaton DE, Bombardier C, Guillemin F, Ferraz MB (2000). Guidelines for the process of cross-cultural adaptation of self-report measures. Spine (Phila Pa 1976).

[47] S. Evidence Summary. Chronic Disease: Self-Management. JBI-ES-1295-3 [Internet]. JBI-ES-1295-3. 2021.

