# Supplementary material for: Appropriateness of exercise therapy delivery in chronic low back pain management: cross-sectional online survey of physiotherapy practice in Germany
Source: BMC Musculoskelet Disord. 2024 May 29;25:422. doi: 10.1186/s12891-024-07505-y (PMC11137918; doi:10.1186/s12891-024-07505-y)
Supplement: Supplementary file 2 — Supplementary Material 2. Appendix II [file 12891_2024_7505_MOESM2_ESM.pdf]

Studieninformation

Aktuelle Praxis der Bewegungstherapie bei chronischen Kreuzschmerzen in der physiotherapeutischen Versorgung in Deutschland

(Online Umfrage)

Liebe/r Teilnehmer/in,

Sie sind Physiotherapeut*in und üben Ihren Beruf derzeit in Deutschland aus? Dann laden wir Sie herzlich zur Teilnahme an dieser Umfrage ein. Bitte lesen Sie sich die folgende Information sorgfältig durch. Sie können dann entscheiden, ob Sie teilnehmen möchten.

Welches Ziel verfolgt die Umfrage?

Wir als Zentrum für Versorgungsforschung der Medizinischen Hochschule Brandenburg haben als Ziel, die Gesundheitsversorgung in Deutschland besser zu verstehen. Im Rahmen dieser Umfrage möchten wir verstehen, wie Bewegungstherapie bei unspezifischen, chronischen Kreuzschmerzen in physiotherapeutischen Einrichtungen in Deutschland durchgeführt wird. Bitte berichten Sie uns, wie Sie Menschen mit unspezifischen, chronischen Kreuzschmerzen bewegungstherapeutisch begleiten.

Wie läuft die Umfrage ab?

Die Teilnahme an der Umfrage dauert einmalig ca. 15 Minuten. Sie ist anonym, d.h. Ihre getätigten Angaben werden ohne personenbezogene Daten gespeichert und verarbeitet. Es sind somit keine Rückschlüsse auf Ihre Person möglich.

Haben Sie einen persönlichen Nutzen?

Sie tragen durch Ihre Teilnahme zu einem verbesserten Verständnis über den Ablauf der physiotherapeutischen Versorgung von Menschen mit chronischen, unspezifischen Kreuzschmerzen bei. Sie selbst werden durch Ihre Teilnahme an dieser Umfrage keinen direkten Nutzen haben. Die Ergebnisse werden wissenschaftlich veröffentlicht und werden u.a. nach Abschluss des Projektes hier zu finden sein: https://www.mhb-fontane.de/zvf-bb.html

Freiwilligkeit und Rücktritt

Die Teilnahme an der Umfrage ist freiwillig. Falls Sie teilnehmen möchten, lesen Sie bitte die beiliegende Einwilligungserklärung und bestätigen ihr Einverständnis über einen Haken im dafür vorgesehenen Kontrollkasten. Da die Befragung anonym stattfindet, können einmal getätigte Angaben nicht mehr zurückgezogen werden. Sie können jederzeit die Befragung vorzeitig beenden. Ihre bis dahin getätigten Angaben werden nicht gespeichert.

Bitte beachten Sie außerdem, dass jegliche Form der Speicherung und Vervielfältigung des Fragebogens auch in Teilen untersagt ist und rechtlich verfolgt wird.

Vielen Dank für Ihre Unterstützung!

Version 2.3 final
17.04.2023

Einwilligungserklärung

Ich habe die Informationsschrift gelesen und habe keine weiteren Fragen. Ich versichere, die beschriebenen Einschlusskriterien zur Teilnahme an dieser Befragung nach bestem Wissen erfülle. Ich versichere außerdem, nur einmalig an der Befragung teilzunehmen.

Datenschutz

Mir ist bekannt, dass bei dieser Studie selbst berichtete Daten zu meiner beruflichen Praxis verarbeitet werden sollen. Die Verarbeitung der Daten erfolgt nach gesetzlichen Bestimmungen und setzt gemäß Art. 6 Abs. 1 lit. c der Datenschutz-Grundverordnung (DGSVO) folgende Einwilligungserklärung voraus:

Ich werde hiermit darüber aufgeklärt und stimme freiwillig zu, dass meine in der Studie erhobenen Daten, insbesondere Angaben zu meiner beruflichen Praxis, zu den in der Informationsschrift beschriebenen Zwecken dokumentiert, ausgewertet und an die Studienleiter und das Forschungsteam des Zentrums für Versorgungsforschung Brandenburg der Medizinischen Hochschule Brandenburg Theodor Fontane weitergegeben werden. Dritte erhalten keinen Einblick zu meinen getätigten Angaben. Die selbst berichteten Daten zu meiner beruflichen Praxis sind zu jedem Zeitpunkt anonym und erlauben keine Rückschlüsse auf meine Person. Eine Löschung anonymer Daten ist nicht möglich, da die Daten nicht meiner Person zugeordnet werden können. Mir ist bewusst, dass ich bei Interesse Auskunft über entstandene Studienergebnisse einholen kann.

☐	Ich habe die Einwilligungserklärung und Studieninformation gelesen und erkläre mich mit den datenschutzrechtlichen Bestimmungen zum Umgang mit meinen getätigten Angaben einverstanden.
☐	Mit der Teilnahme an dieser Umfrage bestätige ich, dass ich anerkannte/r Physiotherapeut*in bin und in Deutschland als Physiotherapeut*in arbeite.

Bei Fragen oder Anliegen kann ich mich an folgende Person wenden:

Lukas Kühn
Nachwuchsgruppe Versorgungsforschung/Rehabilitationswissenschaften
Zentrum für Versorgungsforschung Brandenburg (ZVF-BB)
Seebad 82/83
15562 Rüdersdorf bei Berlin
E-Mail: lukas.kuehn@mhb-fontane.de

Medizinische Hochschule Brandenburg Theodor Fontane
Fehrbelliner Straße 38
16816 Neuruppin

Allgemeine Hinweise

Vielen Dank für Ihre Zeit, an dieser Online-Umfrage teilzunehmen. Wir möchten Sie darauf hinweisen, dass Sie sich jederzeit dazu entscheiden können, einzelne Fragen der Umfrage nicht zu beantworten. Berücksichtigen sie jedoch, dass eine vollständige Beantwortung aller Fragen für die wissenschaftliche Auswertung und Interpretation von hoher Bedeutung ist.

Bitte beachten Sie beim Ausfüllen folgende **Hinweise:**

- *Sofern Sie eine Frage nicht beantworten möchten, können Sie die entsprechende Frage einfach überspringen. Um Ihre Aussagen aber möglichst umfänglich in unseren Auswertungen berücksichtigen zu können, bitten wir Sie aber darum möglichst alle Fragen zu beantworten.*
- *Sofern Sie die Antwort auf eine Frage im Nachgang ändern möchten, ist Ihnen das nur möglich, solang Sie sich auf der aktuellen Seite der Umfrage befinden*

Vielen Dank für Ihre Teilnahme!

Fallvignette (Teil 1)

Lisa, 35 Jahre alt, wird von ihrer Hausärztin zur Physiotherapie überwiesen, nachdem sie seit 16-18 Wochen unter starken Kreuzschmerzen gelitten hat.

In den letzten Jahren hatte sie nicht mehr die Energie, sich körperlich zu betätigen. Seit Beginn der aktuellen Episode ist sie von ihrer Arbeit als medizinische Fachangestellte krankgeschrieben.

Dies ist die dritte und schlimmste Episode von Kreuzschmerzen, die sie erlebt hat. Bei den vorherigen Episoden verschwanden die Schmerzen spontan.

Der Schmerz ist derzeit auf etwa 50% der schlimmsten erlebten Intensität reduziert. Die Schmerzen stören sie nicht beim Schlafen. Derzeit nimmt sie Paracetamol.

Sie ist sehr besorgt über die Intensität der Schmerzen und befürchtet, dass ihre Rückenprobleme dieses Mal nicht verschwinden werden.

Lisa glaubt, dass sie ihren Rücken immer noch hin und wieder schonen muss. Sie hat Angst, die Schmerzen wieder zu verschlimmern, falls sie etwas aus einer ungünstigen Position heben muss.

Befund von Lisas Hausärztin:

- Neurologischer Befund unauffällig
- Ältere MRT-Aufnahmen zeigen altersbedingte degenerative Veränderungen der Lendenwirbelsäule
- ernsthafte spinale Pathologien wurden ausgeschlossen
- Diagnose: „unspezifische, chronische Kreuzschmerzen“

Die Hausärztin wünscht sich eine physiotherapeutische Maßnahme, die einen bewegungstherapeutischen Ansatz fokussiert und hat deshalb eine Krankengymnastik-Verordnung ausgestellt. Zur Art und Umfang der bewegungstherapeutischen Maßnahme wünscht sie sich ausdrücklich Ihre physiotherapeutische Expertise.

A: Auswahl Bewegungsprogramm

Die gelisteten Bewegungsprogramme stellen wissenschaftlich anerkannte und effektive Therapieansätze bei der Behandlung chronischer Kreuzschmerzen dar.

1	Bitte wählen Sie, welche(s) der gelisteten Programme Sie bei chronischen Kreuzschmerzen grundsätzlich am häufigsten empfehlen. *(Mehrfachantwort möglich)*	Pilates<br>Yoga<br>Graded Activity<br>Widerstandstraining	☐<br>☐<br>☐<br>☐	Motor Control<br>Tai Chi<br>Ausdauertraining<br>Sonstige	☐<br>☐<br>☐<br>☐

Die nachfolgenden Aussagen betreffen das oben beschriebene Fallszenario und den Auswahlprozess einer bewegungstherapeutischen Maßnahme. Kreuzen Sie bitte jeweils an, wie weit die Aussage zutrifft[1].

[1] Scholl I, Kriston L, Dirmaier J, Buchholz A, Härter M. Development and psychometric properties of the Shared Decision Making Questionnaire--physician version (SDM-Q-Doc). Patient Educ Couns. 2012;88(2):284-90.

2	[redacted]					
	Trifft überhaupt nicht zu ☐	Trifft weitgehend nicht zu ☐	Trifft eher nicht zu ☐	Trifft eher zu ☐	Trifft weitgehend zu ☐	Trifft völlig zu ☐
3	[redacted]					
	Trifft überhaupt nicht zu ☐	Trifft weitgehend nicht zu ☐	Trifft eher nicht zu ☐	Trifft eher zu ☐	Trifft weitgehend zu ☐	Trifft völlig zu ☐
4	[redacted]					
	Trifft überhaupt nicht zu ☐	Trifft weitgehend nicht zu ☐	Trifft eher nicht zu ☐	Trifft eher zu ☐	Trifft weitgehend zu ☐	Trifft völlig zu ☐
5	[redacted]					
	Trifft überhaupt nicht zu ☐	Trifft weitgehend nicht zu ☐	Trifft eher nicht zu ☐	Trifft eher zu ☐	Trifft weitgehend zu ☐	Trifft völlig zu ☐
6	[redacted]					
	Trifft überhaupt nicht zu ☐	Trifft weitgehend nicht zu ☐	Trifft eher nicht zu ☐	Trifft eher zu ☐	Trifft weitgehend zu ☐	Trifft völlig zu ☐
7	[redacted]					
	Trifft überhaupt nicht zu ☐	Trifft weitgehend nicht zu ☐	Trifft eher nicht zu ☐	Trifft eher zu ☐	Trifft weitgehend zu ☐	Trifft völlig zu ☐
8	[redacted]					
	Trifft überhaupt nicht zu ☐	Trifft weitgehend nicht zu ☐	Trifft eher nicht zu ☐	Trifft eher zu ☐	Trifft weitgehend zu ☐	Trifft völlig zu ☐
9	[redacted]					
	Trifft überhaupt nicht zu ☐	Trifft weitgehend nicht zu ☐	Trifft eher nicht zu ☐	Trifft eher zu ☐	Trifft weitgehend zu ☐	Trifft völlig zu ☐
10	[redacted]					
	Trifft überhaupt nicht zu ☐	Trifft weitgehend nicht zu ☐	Trifft eher nicht zu ☐	Trifft eher zu ☐	Trifft weitgehend zu ☐	Trifft völlig zu ☐

Fallvignette (Teil 2)

Sie haben sich mit Lisa auf ein Bewegungsprogramm geeinigt, das im Kern Widerstandstraining fokussiert. Sie haben mit Lisa außerdem folgende Behandlungsziele vereinbart:

- Verbesserung der allgemeinen körperlichen Funktions- und Leistungsfähigkeit
- Schmerzreduktion und Steigerung der Bewegungstoleranz
- Verbesserung des allgemeinen psychischen Wohlbefindens

Bitte beantworten Sie die folgenden Fragen unter Berücksichtigung des Fallszenarios und der dargestellten Informationen.

B: Dosis Bewegungsprogramm

*Im Folgenden stellen wir Ihnen Fragen zur Häufigkeit und Intensität des Widerstandstrainings. Das vordergründige Ziel soll sein, die Bewegung für Lisa wieder zu normalisieren. Es kommt in erster Linie **nicht** darauf an, vorab definierte Funktionsparameter zu erreichen.*

	Um wirkungsvoll für Lisa zu sein, …				
11	wie häufig in der Woche sollte Lisa aus Ihrer Sicht Widerstandstraining mindestens ausüben?	1x pro Woche<br>2x pro Woche<br>3x pro Woche<br>7x pro Woche	☐<br>☐<br>☐<br>☐	4x pro Woche<br>5x pro Woche<br>6x pro Woche	☐<br>☐<br>☐
12	wie hoch sollte aus Ihrer Sicht die, von Lisa wahrgenommene Intensität des Widerstandstrainings sein?	6 sehr, sehr leicht<br>7<br>8<br>9 sehr leicht<br>10<br>11 leicht<br>12<br>13 etwas anstrengend	☐<br>☐<br>☐<br>☐<br>☐<br>☐<br>☐<br>☐	14<br>15 anstrengend<br>16<br>17 sehr schwer<br>18<br>19 sehr, sehr schwer<br>20	☐<br>☐<br>☐<br>☐<br>☐<br>☐<br>☐<br>☐
13	wie viele Minuten sollte aus Ihrer Sicht eine Trainingseinheit mindestens umfassen?	15 Minuten<br>45 Minuten<br>90 Minuten	☐<br>☐<br>☐	30 Minuten<br>60 Minuten	☐<br>☐
14	über welchen Zeitraum sollte aus Ihrer Sicht die Bewegungstherapie mindestens ausgeübt werden?	4 Wochen<br>12 Wochen<br>48 Wochen	☐<br>☐<br>☐	6 Wochen<br>24 Wochen	☐<br>☐

C: Bewegungsförderung

C1: Mit den nachfolgenden Aussagen möchten wir untersuchen, wie Sie zu den Schmerzen Ihrer Rückenschmerzpatienten wie Lisa stehen. Geben Sie bitte an, in welchem Maß Sie der vorgegebenen Aussage zustimmen[2].

15	[redacted]

[2] Laekeman MA, Sitter H, Basler HD. The Pain Attitudes and Beliefs Scale for Physiotherapists: psychometric properties of the German version. Clin Rehabil. 2008 Jun;22(6):564-75

	Gar nicht einverstanden	Größtenteils nicht einverstanden	Teilweise nicht einverstanden	Teilweise einverstanden	Größtenteils einverstanden	Völlig einverstanden
	☐	☐	☐	☐	☐	☐
16	[redacted]					
	Gar nicht einverstanden	Größtenteils nicht einverstanden	Teilweise nicht einverstanden	Teilweise einverstanden	Größtenteils einverstanden	Völlig einverstanden
	☐	☐	☐	☐	☐	☐
17	[redacted]					
	Gar nicht einverstanden	Größtenteils nicht einverstanden	Teilweise nicht einverstanden	Teilweise einverstanden	Größtenteils einverstanden	Völlig einverstanden
	☐	☐	☐	☐	☐	☐
18	[redacted]					
	Gar nicht einverstanden	Größtenteils nicht einverstanden	Teilweise nicht einverstanden	Teilweise einverstanden	Größtenteils einverstanden	Völlig einverstanden
	☐	☐	☐	☐	☐	☐
19	[redacted]					
	Gar nicht einverstanden	Größtenteils nicht einverstanden	Teilweise nicht einverstanden	Teilweise einverstanden	Größtenteils einverstanden	Völlig einverstanden
	☐	☐	☐	☐	☐	☐
20	[redacted]					
	Gar nicht einverstanden	Größtenteils nicht einverstanden	Teilweise nicht einverstanden	Teilweise einverstanden	Größtenteils einverstanden	Völlig einverstanden
	☐	☐	☐	☐	☐	☐
21	[redacted]					
	Gar nicht einverstanden	Größtenteils nicht einverstanden	Teilweise nicht einverstanden	Teilweise einverstanden	Größtenteils einverstanden	Völlig einverstanden
	☐	☐	☐	☐	☐	☐
22	[redacted].					
	Gar nicht einverstanden	Größtenteils nicht einverstanden	Teilweise nicht einverstanden	Teilweise einverstanden	Größtenteils einverstanden	Völlig einverstanden
	☐	☐	☐	☐	☐	☐
23	[redacted]					
	Gar nicht einverstanden	Größtenteils nicht einverstanden	Teilweise nicht einverstanden	Teilweise einverstanden	Größtenteils einverstanden	Völlig einverstanden
	☐	☐	☐	☐	☐	☐
24	[redacted]					
	Gar nicht einverstanden	Größtenteils nicht einverstanden	Teilweise nicht einverstanden	Teilweise einverstanden	Größtenteils einverstanden	Völlig einverstanden
	☐	☐	☐	☐	☐	☐
25	[redacted]					
	Gar nicht einverstanden	Größtenteils nicht einverstanden	Teilweise nicht einverstanden	Teilweise einverstanden	Größtenteils einverstanden	Völlig einverstanden

	☐	☐	☐	☐	☐	☐
26	[redacted]					
	Gar nicht einverstanden	Größtenteils nicht einverstanden	Teilweise nicht einverstanden	Teilweise einverstanden	Größtenteils einverstanden	Völlig einverstanden
	☐	☐	☐	☐	☐	☐
27	[redacted]					
	Gar nicht einverstanden	Größtenteils nicht einverstanden	Teilweise nicht einverstanden	Teilweise einverstanden	Größtenteils einverstanden	Völlig einverstanden
	☐	☐	☐	☐	☐	☐
28	[redacted]					
	Gar nicht einverstanden	Größtenteils nicht einverstanden	Teilweise nicht einverstanden	Teilweise einverstanden	Größtenteils einverstanden	Völlig einverstanden
	☐	☐	☐	☐	☐	☐
29	[redacted]					
	Gar nicht einverstanden	Größtenteils nicht einverstanden	Teilweise nicht einverstanden	Teilweise einverstanden	Größtenteils einverstanden	Völlig einverstanden
	☐	☐	☐	☐	☐	☐
30	[redacted]					
	Gar nicht einverstanden	Größtenteils nicht einverstanden	Teilweise nicht einverstanden	Teilweise einverstanden	Größtenteils einverstanden	Völlig einverstanden
	☐	☐	☐	☐	☐	☐
31	[redacted]					
	Gar nicht einverstanden	Größtenteils nicht einverstanden	Teilweise nicht einverstanden	Teilweise einverstanden	Größtenteils einverstanden	Völlig einverstanden
	☐	☐	☐	☐	☐	☐

**C2**	*Im Folgenden lesen Sie allgemeine Aussagen zur Entstehung von Schmerzen. Bitte beziehen Sie dazu Stellung*[3].	Richtig	Falsch	Unent-schlossen
32	[redacted]			
33	[redacted]			
34	[redacted]			
35	[redacted]			
36	[redacted]			

[3] Richter M, Maurus B, Egan Moog M. Die deutsche Version des Neurophysiology of Pain Questionnaire. Der Schmerz. 2019;33:244-52.

37	[redacted]			
38	[redacted]			
39	[redacted]			
40	[redacted]			
41	[redacted]			
42	[redacted]			
43	[redacted]			

C3: Selbstmanagement

*Im Folgenden möchten wir feststellen, inwiefern Sie Maßnahmen in Ihrem klinischen Alltag nutzen, die Patient*innen wie Lisa dabei helfen, mit ihrem Gesundheitsproblem umzugehen. Beziehen Sie deshalb bitte Stellung zu den folgenden Aussagen:*

	Um bewegungstherapeutische Maßnahmen bei Patient*innen wie Lisa auch außerhalb der Therapiesitzungen zu fördern…	Nie	Selten	Teils, Teils	Oft	Immer
44	… vereinbaren Sie mit ihr einen Aktionsplan[1].<br>[1]*Eine informelle Vereinbarung von Handlungs- und Bewältigungsstrategien, die außerhalb der Therapiesitzungen Anwendung finden.*	☐	☐	☐	☐	☐
45	…dokumentieren Sie den Aktionsplan schriftlich.	☐	☐	☐	☐	☐
46	… stellen Sie Lisa eine Kopie des Aktionsplans bereit.	☐	☐	☐	☐	☐
47	… überprüfen Sie regelmäßig, ob der Aktionsplan von Lisa umgesetzt wird.	☐	☐	☐	☐	☐
	Anhand vorab definierter Parameter überprüfen Sie regelmäßig, …	Nie	Selten	Teils, teils	Oft	Immer
48	…ob Lisa ihren persönlichen Zielen näher kommt.	☐	☐	☐	☐	☐
49	…ob Lisas Bewegungsprogramm angepasst werden muss.	☐	☐	☐	☐	☐
	Die dafür relevanten Parameter…	Nie	Selten	Teils, teils	Oft	Immer
50	… dokumentieren Sie schriftlich.	☐	☐	☐	☐	☐
51	… stellen Sie Lisa in Form einer Kopie bereit.	☐	☐	☐	☐	☐
	Sie geben Lisa Hilfsmittel *(z.B. Bewegungstagebücher*) an die Hand, …	Nie	Selten	Teils, teils	Oft	Immer

52	…mit denen sie ihre persönlichen Ziele dokumentieren kann.	☐	☐	☐	☐	☐
53	… mit denen sie Fortschritte zu ihren persönlichen Zielen dokumentieren kann.	☐	☐	☐	☐	☐
54	… mit denen sie ihre körperlichen Zeichen und Symptome überwachen kann.	☐	☐	☐	☐	☐

D – Soziodemographie

55	Wie alt sind Sie?	|__|__|__|__|__| (Alter in Jahren)			
56	Welches Geschlecht haben Sie?	Weiblich<br>Divers	☐<br>☐	Männlich	☐
57	In welcher Einrichtungsform arbeiten Sie?	Ambulante Praxis<br>Rehabilitationsklinik	☐<br>☐	Krankenhaus<br>Andere	☐<br>☐
58	In welchem Bundesland arbeiten Sie?	Baden Württemberg<br>Berlin<br>Bremen<br>Hessen<br>Niedersachsen<br>Rheinland-Pfalz<br>Sachsen<br>Schleswig Holsten	☐<br>☐<br>☐<br>☐<br>☐<br>☐<br>☐<br>☐	Bayern<br>Brandenburg<br>Hamburg<br>Mecklenburg-Vorpommern<br>Nordrhein-Westfalen<br>Saarland<br>Sachsen-Anhalt<br>Thüringen	☐<br>☐<br>☐<br>☐<br>☐<br>☐<br>☐<br>☐
59	Welches Arbeitsverhältnis beschreibt Ihre aktuelle Tätigkeit am besten?	angestellt<br>freiberuflich	☐<br>☐	selbstständig	☐
60	Wie sieht Ihr aktuelles Anstellungsverhältnis aus?<br>*(Bei selbstständig, bitte „Nicht zutreffend“ auswählen)*	Unbefristete Anstellung<br>Nicht zutreffend	☐<br>☐	Befristete Anstellung	☐
61	Wie viele physiotherapeutische Kolleg*innen arbeiten außerdem in Ihrem Team?	|__|__| (Bitte Anzahl Kolleg*innen eintragen)			
62	Wie viele Stunden arbeiten Sie in der Woche als klinisch tätige/r Physiotherapeut*in?	|__|__| (Bitte Anzahl der Stunden eintragen)			

63	Wo haben Sie Ihre berufliche Ausbildung absolviert? *(Mehrfachantwort möglich)*	Berufsfachschule<br>Sonstige	☐<br>☐	Hochschule	☐
64	Welchen Abschluss haben Sie im Rahmen Ihrer physiotherapeutischen Ausbildung erworben? *(Bitte nennen Sie nur den höchsten Abschluss)*	Examinierte/r Physiotherapeut*in<br>Bachelor	☐<br>☐	Master<br>Doktorgrad	☐<br>☐
65	Haben Sie eine oder mehrere der folgenden Fortbildungen abgeschlossen? *(Mehrfachantwort möglich)*	Manuelle Therapie (MT)<br>Heilpraktiker*in<br>Schmerzphysiotherapie<br>Sonstige	☐<br>☐<br>☐<br>☐	Orthopädisch manipulative Therapie (OMT)<br>Osteopathie<br>Krankengymnastik am Gerät (KGG)	☐<br>☐<br>☐
66	Wie viele Jahre arbeiten Sie bereits als Physiotherapeut*in?	|__|__| (Bitte Anzahl in Jahren eintragen)			
67	Wie viele Patient*innen mit chronischen, unspezifischen Kreuzschmerzen behandeln Sie in einer typischen Arbeitswoche?	|__|__| (Bitte Schätzung Patientenanzahl eintragen)			
68	Wie viele Patient*innen mit chronischen unspezifischen Kreuzschmerzen werden in Ihrer Einrichtung in einer typischen Arbeitswoche behandelt?	|__|__| (Bitte Schätzung Patientenanzahl eintragen)			
69	Mit welchen, der folgenden Fachgruppen haben Sie in Ihrer täglichen Arbeit regelmäßigen Austausch? *(Mehrfachantwort möglich)*	Medizin<br>Gesundheits- und Krankenpflege<br>Sonstige	☐<br>☐<br>☐	Psychologie<br>Ergotherapie<br>Keinen Austausch	☐<br>☐<br>☐
70	Sind Sie Mitglied in einem physiotherapeutischen Berufsverband oder einer physiotherapeutischen Gesellschaft?	Ja	☐	Nein	☐

71	Wie viel Zeit steht Ihnen an einem normalen Arbeitstag für den Erstbefund zur Verfügung?	|__|__|__| (Anzahl in Min.)
72	Wie viel Zeit steht Ihnen an einem normalen Arbeitstag für eine Patientenbehandlung zur Verfügung?	|__|__|__| (Anzahl in Min.)
73	Über welche Informationsquelle(n) halten Sie sich bei der Behandlung chronischer Kreuzschmerzen auf dem aktuellen Stand? *(Mehrfachantwort möglich)*	Wissenschaftliche Fachzeitschriften ☐<br>Leitlinienempfehlungen wissenschaftlich-medizinischer Fachgesellschaften ☐<br>Informationsquellen von Berufsverbänden ☐<br>Erfahrungsaustausch mit Kolleg*innen ☐<br>Weiterbildungen ☐<br>Sonstige ☐

74	Wie bewerten Sie Ihre eigene Expertise bei der Behandlung chronischer Kreuzschmerzen im Vergleich zu Ihren Berufskolleg*innen in Deutschland?							
	☐ Stark unter-durchschnittlich	☐	☐	☐	☐	☐	☐	☐ Stark über-durchschnittlich
75	Wie bewerten Sie die aktuelle Qualität der durchschnittlichen physiotherapeutischen Grundausbildung in Deutschland?							
	☐ Sehr niedrig	☐	☐	☐	☐	☐	☐	☐ Sehr hoch
76	Wie bewerten Sie die aktuelle Qualität der durchschnittlichen physiotherapeutischen Weiterbildungsangebote in Deutschland?							
	☐ Sehr niedrig	☐	☐	☐	☐	☐	☐	☐ Sehr hoch

Herzlichen Dank für Ihre Teilnahme!
