# Supplementary material for: Appropriateness of exercise therapy delivery in chronic low back pain management: cross-sectional online survey of physiotherapy practice in Germany
Source: BMC Musculoskelet Disord. 2024 May 29;25:422. doi: 10.1186/s12891-024-07505-y (PMC11137918; doi:10.1186/s12891-024-07505-y)
Supplement: Supplementary file 3 — Supplementary Material 3. Appendix III [file 12891_2024_7505_MOESM3_ESM.docx]

**Graphical examination of normal distribution of residuals**


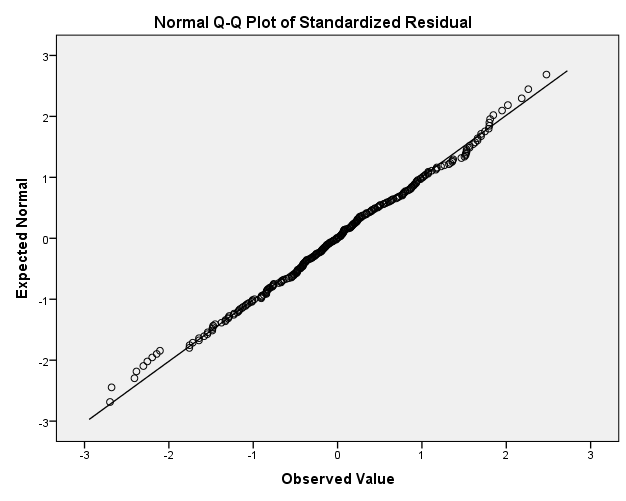


**Figure 1:** Q-Q-plot of standardized residuals


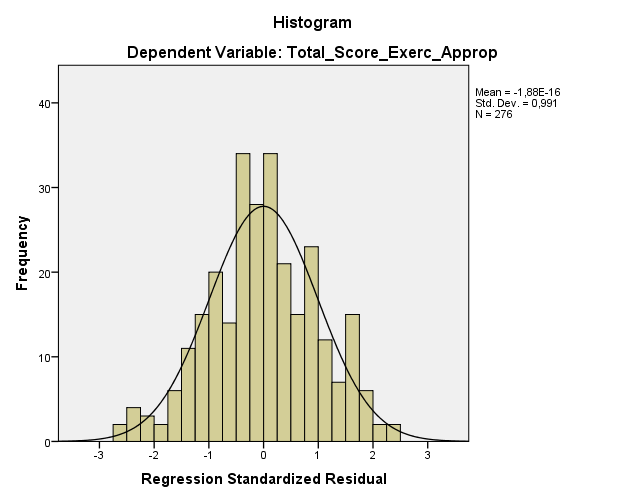


**Figure 2:** Histogram of standardized residuals

**Graphical examination of homoscedasticity**


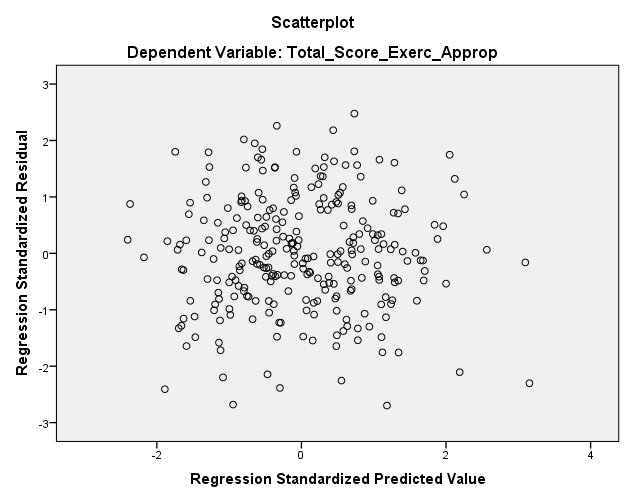


**Figure 3:** Scatterplott of standardized residuals
